# Unexpected severe consequences of *Pikfyve* deletion by aP2‐ or Aq‐promoter‐driven Cre expression for glucose homeostasis and mammary gland development

**DOI:** 10.14814/phy2.12812

**Published:** 2016-06-07

**Authors:** Ognian C. Ikonomov, Diego Sbrissa, Khortnal Delvecchio, James A. Rillema, Assia Shisheva

**Affiliations:** ^1^ Department of PhysiologyWayne State University School of MedicineDetroitMichigan

**Keywords:** Adipose PIKfyve knockout, glucose homeostasis, pregnancy‐induced mammary gland development

## Abstract

Systemic deficiency of PIKfyve, the evolutionarily conserved phosphoinositide kinase synthesizing cellular PtdIns5P and PtdIns(3,5)P_2_ and implicated in insulin signaling, causes early embryonic death in mice. In contrast, mice with muscle‐specific *Pikfyve* disruption have normal lifespan but exhibit early‐age whole‐body glucose intolerance and muscle insulin resistance, thus establishing the key role of muscle PIKfyve in glucose homeostasis. Fat and muscle tissues control postprandial glucose clearance through different mechanisms, raising questions as to whether adipose *Pikfyve* disruption will also trigger whole‐body metabolic abnormalities, and if so, what the mechanism might be. To clarify these issues, here we have characterized two new mouse models with adipose tissue disruption of *Pikfyve* through Cre recombinase expression driven by adipose‐specific aP2‐ or adiponectin (Aq) promoters. Whereas both mouse lines were ostensibly normal until adulthood, their glucose homeostasis and systemic insulin sensitivity were severely dysregulated. These abnormalities stemmed in part from accelerated fat‐cell lipolysis and elevated serum FFA. Intriguingly, aP2‐Cre‐PIKfyve^fl/fl^ but not Aq‐Cre‐PIKfyve^fl/fl^ females had severely impaired pregnancy‐induced mammary gland differentiation and lactogenesis, consistent with aP2‐Cre‐mediated *Pikfyve* excision in nonadipogenic tissues underlying this defect. Intriguingly, whereas mammary glands from postpartum control and Aq‐Cre‐PIKfyve^fl/fl^ mice or ex vivo mammary gland explants showed profound upregulation of PIKfyve protein levels subsequent to prolactin receptor activation, such increases were not apparent in aP2‐Cre‐PIKfyve^fl/fl^ females. Collectively, our data identify for the first time that adipose tissue *Pikfyve* plays a key role in the mechanisms regulating glucose homeostasis and that the PIKfyve pathway is critical in mammary epithelial differentiation during pregnancy and lactogenesis downstream of prolactin receptor signaling.

## Introduction

Mounting evidence implicates the enzymes catalyzing synthesis and turnover of phosphoinositides (PI) in human diseases, such as diabetes, cancer, neurological disorders, and others (Di Paolo and De Camilli 2006; Yuan and Cantley 2008; Hakim et al. 2012; Mayinger 2012; Shisheva 2012; Balla 2013; Sun et al. 2013). Although most mammalian PI‐metabolizing enzymes have a number of isoforms with a redundant function, the evolutionarily conserved PIKfyve (PhosphoInositide Kinase for position five containing a fyve domain) (gene symbol *PIKFYVE*), responsible for synthesis of nearly the entire cellular amounts for two of the seven PIs, that is, PtdIns5P and PtdIns(3,5)P_2_, is a product of a single gene (Shisheva 2008b). The low‐abundance PtdIns5P and PtdIns(3,5)P_2_ are signaling lipids, regulating various aspects of essential cellular processes such as endomembrane homeostasis, endosome processing in the course of membrane trafficking, insulin‐regulated cytoskeletal dynamics, autophagy, nuclear transport, gene transcription, and cell cycle (Shisheva 2012; Viaud et al. 2014; Shisheva et al. 2015; Vicinanza et al. 2015). The nonredundant function of PIKfyve is evident by the early embryonic lethality of *Pikfyve* null mice, occurring at the preimplantation (Ikonomov et al. 2011) or postimplantation stage (Takasuga et al. 2013). The phenotype severity of the systemic *Pikfyve* disruption is proportional to the level of the remaining protein/activity. For example, genetically modified mice expressing ~10% residual PIKfyve survive up to 2 weeks after birth, having various defects in several tissues, whereas heterozygous PIKfyve^−/+^ mice that exhibit ~50% residual protein/activity in all tissues, including brain, have a normal lifespan without ostensible tissue/organ abnormalities (Shisheva et al. 2015). In humans, the *PIKFYVE* mutations identified thus far are all heterozygous and outside the catalytic domain, triggering only mild corneal dystrophy (Li et al. 2005). Thus, combined data in humans and genetically modified mice suggest that key mutations in the *PIKFYVE* gene might be incompatible with human life, leading, presumably, to a demise of the developing embryo. Furthermore, consistent with the requirement for a tight control in PtdIns(3,5)P_2_/PtdIns5P production, PIKfyve forms a multiprotein complex incorporating the phosphatidylinositol 5‐phosphatase Sac3 (Sac1 domain‐containing phosphoinositide 5‐phosphatase 3, gene symbol *FIG4*) that not only turns over PtdIns(3,5)P_2_/PtdIns5P but also activates PIKfyve (Sbrissa et al. 2007; Ikonomov et al. 2009a). Concordantly, mutations in Sac3 are linked to human disease (Lenk and Meisler 2014).

PIKfyve was first identified in the context of muscle and fat tissues, both of which harbor the insulin‐regulated glucose transporter GLUT4 (Shisheva et al. 1999). The role of PIKfyve and its lipid products as part of the signaling cascade triggering insulin‐stimulated glucose transport and GLUT4 cell‐surface accumulation was subsequently confirmed in cultured 3T3L1 adipocytes and primary fat cells (Ikonomov et al. 2007, 2009b; Koumanov et al. 2012). Mechanistically, PIKfyve functions downstream of the PI3Ks that produce, directly or indirectly, its enzymatic substrate, that is, PtdIns3P, therefore, processes controlled by PIKfyve signaling would require normal PI3K (phosphoinositide 3‐kinase) functioning (Shisheva 2012; Ikonomov et al. 2015). Recent data in genetically modified mice (MPIfKO) with PIKfyve deficiency localized specifically in skeletal muscle, the tissue responsible for the majority of insulin‐regulated postprandial glucose disposal (Biddinger and Kahn 2006), has provided the first in vivo evidence for the central role of muscle PIKfyve in the mechanisms regulating whole‐body glucose homeostasis (Ikonomov et al. 2013). The MPIfKO mice exhibited systemic glucose intolerance and impaired insulin‐induced glucose uptake in muscle as well as increased adiposity and hyperinsulinemia but not dyslipidemia (Ikonomov et al. 2013). Thus, the combined phenotype manifested by the MPIfKO mouse recapitulates the cluster of typical features in clinical insulin resistance, also known as prediabetes, that includes systemic glucose intolerance and insulin resistance, hyperinsulinemia, and increased visceral obesity without dyslipidemia (Abdul‐Ghani and DeFronzo 2009).

The direct role of adipose tissue to the postprandial glucose clearance is minor (Biddinger and Kahn 2006; Herman and Kahn 2006). Nevertheless, it contributes to the development of prediabetes and T2D due to a failure of its two key functions: to store lipids as TG and to secrete bioactive factors (Yu and Ginsberg 2005; Jensen 2006; Deng and Scherer 2010). The current study was designed to explore for the first time the significance of adipose PIKfyve in glucose homeostasis. To this end, we initially crossed our PIKfyve^fl/fl^ mice (Ikonomov et al. 2011) with a mouse line expressing Cre recombinase driven by the widely used 5.4 kb promoter/enhancer of the *aP2/FABP4* (adipose protein 2/fatty acid binding protein 4) gene (He et al. 2003). Whereas we found that the PIKfyve^fl/fl,aP2‐Cre+^ mice of both genders had dysregulated glucose homeostasis, the females also exhibited severe defects in mammary gland differentiation during pregnancy and in lactogenesis, causing early postnatal death of the offspring in primiparity or multiparity. To further explore the role of adipose PIKfyve in glucose homeostasis as well as to reveal if PIKfyve‐deficient adipose tissue underlies the striking mammary phenotype, we generated another fat tissue‐specific mouse line, PIKfyve^fl/fl,Aq‐Cre+^, in which Cre‐recombinase is driven by the promoter of the most adipocyte‐specific gene, that is, adiponectin (Wang et al. 2010; Eguchi et al. 2011). Although PIKfyve^fl/fl,Aq‐Cre+^ mice exhibited severely impaired glucose homeostasis, the mammary gland phenotype was not reproduced, consistent with aP2‐Cre‐mediated *Pikfyve* excision in nonadipogenic tissues causing the impaired mammogenesis and defective lactogenesis. Furthermore, in search of the mechanisms underlying impaired glucose homeostasis in PIKfyve^fl/fl,Aq‐Cre+^ mice, we established accelerated fat‐cell lipolysis and elevated serum FFA (free fatty acids), thus revealing an unexpected role of adipose *Pikfyve* in the mechanisms regulating fat tissue TG storage.

## Methods

### Ethical approval

Animal numbers and all animal procedures were approved by the Wayne State University Animal Care and Use Committee (IACUC). The study was conducted according to institutional ethics guidelines for animal work by the Association and Accreditation of Laboratory Animal Care.

### PIKfyve^fl/fl,aP2‐Cre+^ and PIKfyve^fl/fl,Aq‐Cre+^ mice

The development of conditional PIKfyve^fl/fl^ mice on a C57BL/6J background by the Cre‐loxP approach was described elsewhere (Ikonomov et al. 2011). To generate mice with selective disruption of *Pikfyve* in adipose tissue, the PIKfyve^fl/fl^ progeny was crossed with mice expressing Cre recombinase under the adipose tissue‐specific promoters of aP2 (generated by Ronald Evans and purchased from The Jackson Laboratory; B6.Cg‐Tg(Fabp4‐cre)1Rev/J) and of Adiponectin (a kind gift by Dr. Evan Rosen, supplied by Dr. Richard M Mortensen on a C57BL/6J background). Genotyping was performed by PCR using genomic DNA isolated from tails of 20‐day‐old mice and specific primer‐pairs detailed previously (Ikonomov et al. 2011). PIKfyve^fl/fl,aP2‐Cre+^ and PIKfyve^fl/fl,Aq‐Cre+^ mice were used as homozygous mutants; PIKfyve^fl/fl^ mice of the respective breeding were used as controls and PIKfyve^fl/wt,aP2‐Cre+^ or PIKfyve^fl/wt,Aq‐Cre+^ were used as heterozygous mutants. Most of the experiments were performed by comparing PIKfyve^fl/fl^ controls versus the respective PIKfyve^fl/fl,aP2‐Cre+^ and PIKfyve^fl/fl,Aq‐Cre+^ mice. Animals were maintained in a temperature‐controlled environment (22 ± 1°C) with a 12‐h light/dark cycle (6 am/6 pm) and a standard rodent diet (4.5% calories from fat; LabDiets #5001) with free access to food and water. Separate groups of male and female mice were used as indicated.

### Body composition and blood/serum metabolites

Fat and lean mass was measured in randomly fed nonanesthetized mice, typically between noon and 2 pm, using EcoMRI (Echo Medical systems 130). Glucose was determined by a glucometer (Truetrack) in a blood droplet formed after mouse tail clipping. Nonesterified fatty acids (FFA) and glycerol concentrations were measured in serum of 4 h fasted animals using enzymatic kits from Wako (99475409, Richmond, VA) and Sigma (TR0100 kit, Saint Louis, MO). Adiponectin was evaluated in serum by WB. Serum was derived from blood collected by heart puncture after mouse euthanasia by cervical dislocation.

### Glucose and insulin tolerance tests

For GTT (glucose tolerance test) and ITT (insulin tolerance test), individually housed (on paper bedding), and morning‐fasted mice (5 h) were injected intraperitoneally (i.p.) with 1.5 g dextrose/kg body weight or insulin (0.75 U/kg body weight) (Humalog, Lilly). Tail blood prior to, and following glucose or insulin injections was used for glucose measurements at the indicated time intervals. Excel (Microsoft Inc.) and the trapezoid rule were used to calculate the area under the curve (AUC) from time zero to the last time point (120 min in GTT and 60 min in ITT) postinjection as previously described (Ikonomov et al. 2013).

### In vivo insulin stimulation

Following 14 h fasting, mice were injected i.p. with either saline or 5 U/kg body weight of insulin. Five‐min postinjections mice were sacrificed by cervical dislocation. Adipose tissue was removed and frozen in liquid N_2_ for immunoblot analyses of the insulin signaling proteins.

### Fat cell isolation and lipolysis

Fat cells from freshly isolated perigonadal fat were prepared by collagenase (type I; 134 unit/mg) digestion in KRH (Krebs‐Ringer‐Hepes) buffer, pH 7.4, as we previously described (Shisheva and Shechter 1992, 1993). Briefly, duplicate samples of fat cell suspension (~3 × 10^5^ cells/mL, in KRH buffer containing 0.7% BSA) were treated with isoproterenol (25 μmol/L) (Sigma) for 60 min at 37°C. When insulin (human recombinant, a gift by Eli Lilly) was present, it was added 5 min prior to the incubation with isoproterenol. At the end of the incubation, glycerol was measured enzymatically (TR0100 kit from Sigma, Saint Louis, MO) at 540 nm in duplicate aliquots taken from the aqueous phase of the reaction mix.

### Antibodies and Western blotting analyses

Rabbit polyclonal anti‐PIKfyve (R7069; East‐Acres, MA) (Sbrissa et al. 1999), anti‐ArPIKfyve (Associated regulator of PIKfyve) (WS047; Covance, Denver, PA) (Sbrissa et al. 2004a), anti‐Sac3 (Sbrissa et al. 2007) and anti‐GDI1 (GDP dissociation inhibitor 1) antibodies (Shisheva et al. 1994) (R5057; East‐Acres, MA) were produced and characterized previously. Anti‐phosphoThr308‐Akt (#9275), anti‐phosphoSer473‐Akt (#9271), anti‐Akt, and anti‐phosphoTyr694‐Stat5 (#4322) antibodies were from Cell Signaling (Beverly, MA). Polyclonal antibodies recognizing both Stat5a and Stat5b (Signal transducer and activator of transcription 5) were from Santa Cruz Biotechnology (sc‐835). Anti‐adiponectin antibodies were a kind gift by Dr. Phil Scherer. Western blotting was conducted as described (Ikonomov et al. 2011). Briefly, harvested mouse tissue was homogenized in RIPA^+^ (radioimmuoprecipitation assay) buffer (50 mmol/L Tris/HCl buffer, pH 8.0, containing 150 mmol/L NaCl, 1% Nonidet P‐40, 0.5% Na deoxycholate), supplemented with 1 × protease inhibitor mixture (1 mmol/L phenylmethylsulphonylfluoride, 5 *μ*g/mL leupeptin, 5 *μ*g/mL aprotinin, 1 *μ*g/mL pepstatin, and 1 mmol/L benzamidine) or RIPA^2+^ buffer, containing in addition 1 × phosphatase inhibitor mixtures (50 mmol/L NaF, 10 mmol/L Na pyrophosphate, 25 mmol/L Na *β*‐glycerophosphate and 2 mmol/L Na metavanadate). Lysate protein was resolved by SDS‐PAGE, transferred onto nitrocellulose membrane and probed with the indicated antibodies.

### Immunoprecipitation and in vitro lipid kinase activity

Mouse tissue lysates, clarified by centrifugation, were subjected to immunoprecipitation with anti‐PIKfyve antibodies as previously described (Ikonomov et al. 2013). Immunoprecipitates were subjected to Western blotting or to in vitro PIKfyve lipid kinase assays followed by TLC (thin layer chromatography) lipid resolution as detailed elsewhere (Ikonomov et al. 2013).

### Tissue histology

Mammary glands (#4 inguinal) were dissected from female mice at postpartum day 3 and fixed in 4% formaldehyde overnight at room temperature. The samples were then refixed in 1:1:1 (v/v) fixative, consisting of aqueous 2% osmium tetroxide, 2.5% glutaraldehyde in 0.1 mol/L phosphate buffer, and 0.2 mol/L Sorenson's phosphate buffer, in an ice bath for 1.5 h, then dehydrated in increasing concentrations of ethanol, and finally infiltrated in Epon‐Araldite plastic as described previously (Li et al. 2014). Thin sections were cut and stained in modified Richardson's stain as specified earlier (Li et al. 2014).

### Mammary gland explants

Cultures of mammary gland explants were prepared from midpregnant (12–14 days of pregnancy) Swiss‐Webster mice (Harlan Laboratories, Indianapolis, IN) as previously described (Rillema 1994). Briefly, inguinal and thoracic mammary glands from 6 to 12 mice were removed, cut into small pieces and placed on siliconized lens paper floating on 6 mL M199 medium, containing insulin (1 *μ*g/mL) and cortisol (10^−7^ mol/L). Following 24 h incubation at 37°C in an atmosphere of 95% O_2_‐5% CO_2_, the explants were treated without or with prolactin (1 *μ*g/mL) for an additional 24 h. RIPA^2+^ lysates were obtained and analyzed by Western blotting with or without immunoprecipitation.

### Statistics

Data from PIKfyve^fl/fl,aP2‐Cre+^ and PIKfyve^fl/fl,Aq‐Cre+^ mice were compared with those obtained for flox/flox littermates or age‐matched mice of the corresponding cross by Student's *t* test. Statistical significance of ITT and GTT curves was determined by comparing differences in AUC of the groups by Student's *t* test. The films from Western blots and TLC were scanned at 300 d.p.i. for quantitative analysis. Several films of different exposure times were scanned and quantified to assure that the signals were within the linear range. All data are presented as mean ± SEM. Statistical significance is considered when *P* < 0.05.

## Results

### Validation of the PIKfyve^fl/fl,aP2‐Cre+^ mouse model

The PIKfyve^fl/fl^ mouse line (Ikonomov et al. 2011) was mated with mice carrying Cre recombinase under the aP2 promoter. Tail‐DNA genotyping of the F1–F3 progeny using PCR primers for Cre or floxed alleles identified mice with PIKfyve^fl/fl,aP2‐Cre+^ and PIKfyve^fl/wt,aP2‐Cre+^ genotypes of both genders for subsequent experimentation. PIKfyve^fl/fl,aP2‐Cre+^ mice were born at the expected Mendelian ratio. They were apparently normal, healthy and fertile, with a life span similar to that of their littermate PIKfyve^fl/fl^ brothers and sisters up to 10 months of age, when the study ended.

**Figure 1:**
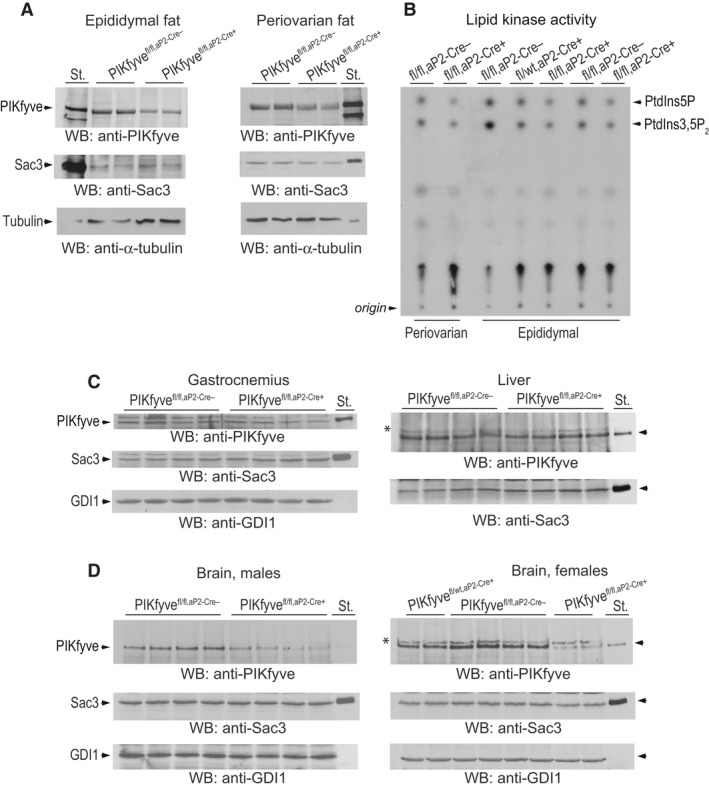
Validation of fat‐specific ablation of PIKfyve in PIKfyve^fl/fl,^ ^aP^ ^2‐Cre+^ mice by Western blotting and in vitro kinase activity. (A) Clarified RIPA ^+^ lysates (100 *μ*g protein), derived from indicated fat depots dissected from PIKfyve^fl/fl,^ ^aP^ ^2‐Cre−^ mice and PIKfyve^fl/fl,^ ^aP^ ^2‐Cre+^ littermates, were examined by Western blotting (WB) with anti‐PIKfyve and Sac3 antibodies with a stripping step in between. Blots were reprobed with anti‐*α*‐tubulin to normalize for loading. Shown are chemiluminescence detections from representative experiments with 2 mice/genotype for each condition out of three independent determinations. (B) Clarified fresh RIPA ^+^ lysates (600 *μ*g protein), derived from the indicated fat dissected from PIKfyve^fl/fl,^ ^aP^ ^2‐Cre+^, PIKfyve^fl/wt,^ ^aP^ ^2‐Cre+^, and PIKfyve^fl/fl,^ ^aP^ ^2‐Cre−^ sisters underwent immunoprecipitation (IP) with anti‐PIKfyve antibodies. Washed IPs were subjected to in vitro lipid kinase activity assay. Shown is a representative autoradiogram of a TLC plate with resolved radiolabeled lipids, showing that only in the Cre^+^ mice were the PIKfyve lipid products significantly abrogated (arrows). These products were also at significantly lower levels in heterozygotes PIKfyve^fl/wt,^ ^aP^ ^2‐Cre+^ than in control PIKfyve^fl/fl,^ ^aP^ ^2‐Cre−^ mice. *C and D,* Clarified RIPA ^+^ lysates from gastrocnemius (200 *μ*g protein), liver and brain (150 *μ*g protein) dissected from PIKfyve^fl/fl,^ ^aP^ ^2‐Cre+^ and PIKfyve^fl/fl,^ ^aP^ ^2‐Cre−^ littermate mice were subjected to WB with PIKfyve and Sac3 antibodies. No significant changes between control and PIKfyve^fl/fl,^ ^aP^ ^2‐Cre+^ mice were apparent in gastrocnemius and liver, but in brains of both PIKfyve^fl/fl,^ ^aP^ ^2‐Cre+^ males and females PIKfyve, but not Sac3 was drastically decreased. Blots were reprobed with anti‐GDI1 to normalize for loading. Equal loading is also apparent by the identical intensity of the unspecific bands in some tissue samples (*). Shown are chemiluminescence detections from representative experiments with 2 or 4 mice/genotype for each condition out of three independent determinations with similar results. St., PIKfyve or Sac3 standards from lysates of COS7 cells overexpressing these proteins.

**Figure 2:**
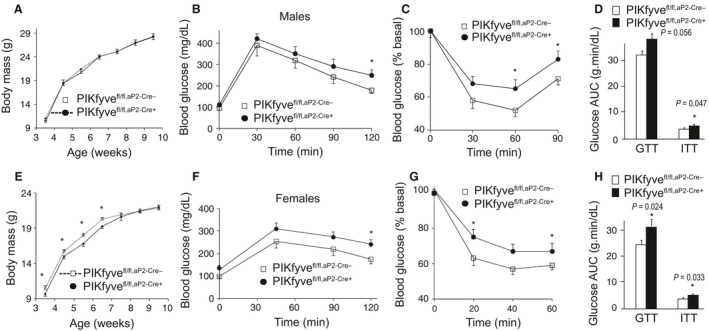
Systemic glucose intolerance and insulin resistance but normal body weight in PIKfyve^fl/fl,^ ^aP^ ^2‐Cre+^ males and females. (A and E): Rate of body weight gain in male (A, *n* = 7/group) and female mice (E, *n* = 7/group) fed a regular diet. *P <* *0.05 versus controls. (B, C, F. and G): Blood glucose levels were measured at indicated time points before and after 1.5 g/kg of glucose (B and F) or 0.75 U/kg of insulin (C and G), administered i.p. in 12–14‐week‐old male and female mice. At this age, the control and mutant mice continued to show lack of significant differences in their body weights. *P <* *0.05 versus control; *n* = 6–8/group. (D and H): The area under the glucose curves (AUC) during GTT and ITT, indicating significant (*P* values are depicted on the graph) insulin resistance in both male and female PIKfyve^fl/fl,^ ^aP^ ^2‐Cre+^ mice.

Western blot analyses with anti‐PIKfyve antibodies revealed that PIKfyve protein levels were decreased by ~50–60% in perigonadal fat pads of both male and female PIKfyve^fl/fl,aP2‐Cre+^ mice versus corresponding PIKfyve^fl/fl^ littermates (Fig. 1A). Considering the confounding effect by other cell types present in fat tissue, the *Pikfyve* gene inactivation was at reasonable efficacy. Data obtained with immunopurified PIKfyve preparations derived from periovarian and epididymal fat also revealed that the PIKfyve enzymatic activity was similarly reduced in PIKfyve^fl/fl,aP2‐Cre+^ mice as evidenced by the selective diminution in in vitro generated PtdIns(3,5)P_2_ and PtdIns5P products (Fig. 1B). Fat tissue of PIKfyve^fl/wt,aP2‐Cre+^ heterozygotes also exhibited reduced synthesis of the two lipid products, though to a lesser extent compared to PIKfyve^fl/fl,aP2‐Cre+^ mice, as expected for haploinsufficiency (Fig. 1B). In contrast, fat‐tissue levels of the PIKfyve‐associated phosphoinositide 5‐phosphatase Sac3 (Sbrissa et al. 2007; Ikonomov et al. 2009a) remained unaltered (Fig. 1A), validating that we monitor solely the consequences of *Pikfyve* inactivation. Notably, whereas in our conditional model, the aP2 promoter did not confer recombination in other metabolic tissue such as muscle and liver (Fig. 1C), there was a pronounced (40–50%) loss of PIKfyve, but not of Sac3, in the brain (Fig. 1D) in agreement with other observations using aP2‐Cre‐driven recombination (Urs et al. 2006; Martens et al. 2010; Heffner et al. 2012; Zhang et al. 2012). This reduction was apparent in both male and female PIKfyve^fl/fl,aP2‐Cre+^ mice and was evident even in brains derived from PIKfyve^fl/wt,aP2‐Cre+^ heterozygotes (Fig. 1D). It should be emphasized, however, that despite the PIKfyve reduction in brain, the PIKfyve^fl/fl,aP2‐Cre+^ mice of both sexes were normal in terms of body weight (Fig. 2A and E), fertility (Table 1), organ morphology, and organ size (not shown). This observation is reminiscent of the heterozygous PIKfyve^WT/KO^ mice with *Pikfyve* gene inactivation at the whole‐body level, which, while exhibiting ~50% reduction in PIKfyve protein or activity in the brain, were ostensibly normal (Ikonomov et al. 2011).

**Table 1:** Early postnatal lethality of the litter born to PIKfyve^fl/fl,aP2‐Cre+^ dams

Female genotype	Primiparas^b^ (*n*)	Litter size	
		At birth (*n*)	96 h postpartum (*n*)
PIKfyve^fl/fl,aP2‐Cre−^	12	6.6 ± 0.6	6.6 ± 0.6
PIKfyve^fl/fl,aP2‐Cre+^	7	6.4 ± 0.4	0.4 ± 0.4^a^
PIKfyve^fl/wt,aP2‐Cre+^	7	6.3 ± 0.5	5.7 ± 0.6
PIKfyve^fl/fl,Aq‐Cre−^	9	6.2 ± 0.1	6.2 ± 0.1
PIKfyve^fl/fl,Aq‐Cre+^	14	6.6 ± 0.3	6.6 ± 0.3

Data are presented as mean ± SEM.

a in a single PIKfyve^fl/fl,aP2‐Cre+^ dam three pups died after the 96th h.

b Data for second and third pregnancies of PIKfyve^fl/fl,aP2‐Cre+^ mice are presented in text.

### PIKfyve^fl/fl,aP2‐Cre+^ mice are glucose intolerant and insulin resistant

Although female PIKfyve^fl/fl,aP2‐Cre+^ mice were slightly lighter at weaning, their body weight rapidly advanced and after 6 weeks, they reached the weight of their PIKfyve^fl/fl^ littermates (Fig. 2E). The growth rate for body weight of male PIKfyve^fl/fl,aP2‐Cre+^ and PIKfyve^fl/fl^ littermate controls were similar (Fig. 2A). To determine the systemic metabolic consequences of PIKfyve‐deficient adipose tissue, we performed glucose‐ and insulin‐tolerance tests (GTT and ITT) in 3‐month‐old male and female PIKfyve^fl/fl,aP2‐Cre+^ mice and compared them with PIKfyve^fl/fl^ sex‐ and age‐matched controls. GTT in male mice revealed that, whereas blood glucose levels at 0 min were similar between the control and mutant groups, they were greater in the PIKfyve^fl/fl,aP2‐Cre+^ mice subsequent to glucose injection (Fig. 2B). Similarly, higher glucose levels during GTT were seen with the female PIKfyve^fl/fl,aP2‐Cre+^ compared to PIKfyve^fl/fl^ littermate mice (Fig. 2F). In both genders, the difference became statistically significant at later time points, consistent with the idea for defective glucose clearance by the muscle (or fat) rather than suppression of endogenous glucose production by the liver (or kidney). Concordantly, the glucose area under the curve (AUC) during GTT for both males and females was larger in the PIKfyve^fl/fl,aP2‐Cre+^ genotype compared to age‐matched controls, reaching statistical significance for the female mice (Fig. 2D and H).

Insulin sensitivity in 3‐month‐old male or female PIKfyve^fl/fl,aP2‐Cre+^ mice was also impaired as judged by the blunted hypoglycemic response to i.p. injection of insulin during ITT. Thus, as illustrated in Fig. 2C and G, blood glucose levels in PIKfyve^fl/fl,aP2‐Cre+^ mice remained higher compared to those in sex‐ and age‐matched control mice at any single time point. Of note, basal glucose levels between the two groups of each sex were not statistically different (not shown). The reduced insulin sensitivity was further underscored by the AUC during ITTs, which was significantly larger in both male and female PIKfyve^fl/fl,aP2‐Cre+^ mice compared to the respective age‐ and sex‐matched control PIKfyve^fl/fl^ mice (Fig. 2D and H). Together, these data demonstrate that both male and female PIKfyve^fl/fl,aP2‐Cre+^ mice exhibit systemic glucose intolerance and insulin resistance.

### Validation of the PIKfyve^fl/fl,Aq‐Cre+^ mouse model

**Figure 3:**
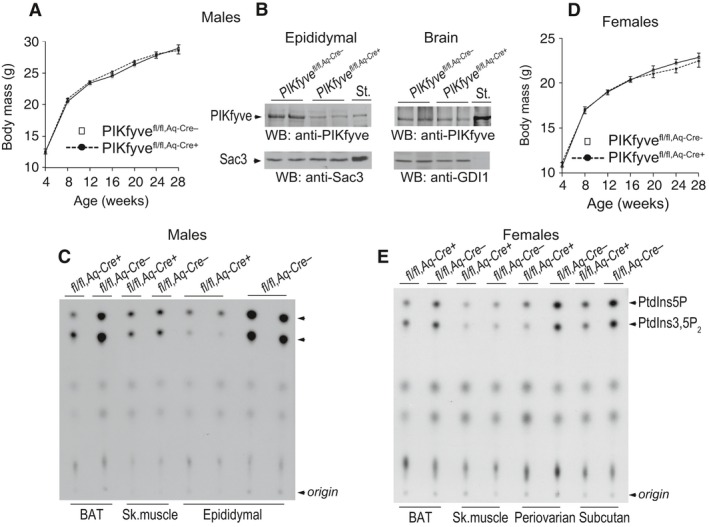
Validation of fat‐specific ablation of PIKfyve in PIKfyve^fl/fl,Aq‐Cre+^ mice by Western blotting or in vitro kinase activity. (A and D) No differences in body weight gain of male (A, *n* = 12/group) or female PIKfyve^fl/fl,Aq‐Cre+^ mice (D, *n* = 7/group) fed a regular diet. (B) Clarified RIPA ^+^ lysates derived from fat (100 *μ*g protein) or brain tissues (150 *μ*g protein) dissected from male PIKfyve^fl/fl,Aq‐Cre+^ and PIKfyve^fl/fl,Aq‐Cre−^ littermate mice, were examined by WB with anti‐PIKfyve, Sac3 or GDI1 (for equal loading) antibodies with a stripping step in between. PIKfyve was profoundly decreased in fat (by ~80%) but not in brain of PIKfyve^fl/fl,Aq‐Cre+^ compared to PIKfyve^fl/fl,Aq‐Cre−^ controls. Shown are chemiluminescence detections from representative experiments with 2 mice/genotype for each tissue out of three to five independent determinations. St., PIKfyve or Sac3 standards from lysates of COS7 cells overexpressing these proteins. (C and E), Clarified fresh RIPA ^+^ lysates (600 *μ*g protein), derived from the indicated tissues dissected from PIKfyve^fl/fl,Aq‐Cre+^ and PIKfyve^fl/fl,Aq‐Cre−^ male (C) or female (E) mice underwent immunoprecipitation (IP) with anti‐PIKfyve antibodies for measuring the in vitro lipid kinase activity. Shown are representative autoradiograms of a TLC plate with resolved radiolabeled lipids demonstrating that in the fat depots of the PIKfyve^fl/fl,Aq‐Cre+^ mice, but not in the other tissues, synthesis of the PIKfyve lipid products were profoundly reduced.

The substantial reduction in PIKfyve levels in brain of both male and female PIKfyve^fl/fl,aP2‐Cre+^ mice suggests the possibility that dysregulated glucose homeostasis could be adversely associated with decreased PIKfyve in brain rather than in adipose tissue. Therefore, to identify the relative contribution of adipocyte PIKfyve to the observed dysregulation of glucose homeostasis as well as to begin exploring the potential mechanisms for the systemic glucose intolerance and insulin resistance, we have generated another fat‐tissue specific *Pikfyve* KO (knockout) model, in which expression of Cre recombinase is driven by the promoter of adiponectin, a gene that is strictly adipose‐specific (Wang et al. 2010; Eguchi et al. 2011; Lee et al. 2013). As PIKfyve^fl/fl,aP2‐Cre+^, the PIKfyve^fl/fl,Aq‐Cre+^ mice were born at the expected Mendelian ratio. They were ostensibly normal, healthy, and fertile (Table 1), with growth curves for body weight (Fig. 3A and D), being similar to those of their littermate PIKfyve^fl/fl^ brothers and sisters up to at least 10 months of age.

There were marked decreases in PIKfyve protein levels as well as in PIKfyve lipid kinase activity (by ~80%) in perigonadal fat pads isolated from both male and female PIKfyve^fl/fl,Aq‐Cre+^ mice versus the corresponding control PIKfyve^fl/fl^ littermates as revealed by Western blotting and in vitro lipid kinase assays (Fig. 3B, C, and E). Levels of the Sac3 phosphatase remained unaltered (Fig. 3B). In other fat depots of both genders, such as BAT or subcutaneous fat, PIKfyve was also profoundly reduced, as illustrated herein by dramatically decreased lipid kinase activity of immunopurified PIKfyve preparations (Fig. 3C and E). Notably, we did not observe any reduction in PIKfyve protein levels in brain of PIKfyve^fl/fl,Aq‐Cre+^ mice (Fig. 3B), nor were there changes in skeletal muscle in both males and females, as measured by the in vitro lipid kinase activity in anti‐PIKfyve immunoprecipitates (Fig. 3C and E).

### PIKfyve^fl/fl,Aq‐Cre+^ mice are glucose intolerant and insulin resistant

**Figure 4:**
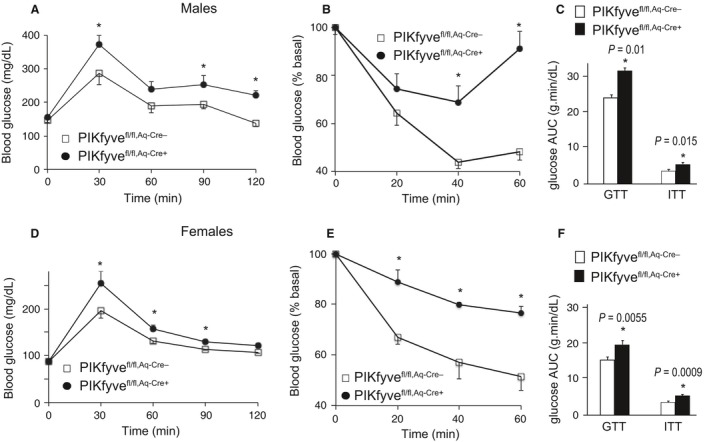
Systemic glucose intolerance and insulin resistance in both PIKfyve^fl/fl,Aq‐Cre+^ males and females. (A, B, D, and E): Blood glucose levels were measured at indicated time points before and after 1.5 g/kg of glucose (A and D) or 0.75 U/kg of insulin (B and E), administered i.p. in 24–26‐week‐old male (*n* = 10–12/group) and female mice (*n* = 6–8/group); *P <* *0.05 versus controls. (C and F): The area under the glucose curves (AUC) during GTT and ITT, showing significant (*P* values are denoted) insulin resistance in both male and female PIKfyve^fl/fl,Aq‐Cre+^ mice.

Both male and female PIKfyve^fl/fl,Aq‐Cre+^ mice exhibited profoundly dysregulated glucose homeostasis and systemic insulin resistance as evidenced by the GTT and ITT in 6‐month‐old mice. Thus, as illustrated in Figure 4A, B, D, and E, glucose levels remained markedly higher subsequent to i.p injection of either glucose or insulin, with statistically significant differences at each time point. In contrast, basal levels of fasting glucose did not statistically differ between the two groups of each sex (Fig. 4A, B, D, and E). Of note, the differences in glucose levels were statistically significant at the early as well as the late time points of GTT in both genders, consistent with the idea that both defective glucose clearance by the muscle (or fat) and suppression of endogenous glucose production by the liver (or kidney) may account for the defects. The profound systemic glucose intolerance and insulin resistance in PIKfyve^fl/fl,Aq‐Cre+^ mice are underscored upon calculating AUC during ITTs and GTTs (Fig. 4C and F). In both male and female PIKfyve^fl/fl,Aq‐Cre+^ mice these values were markedly greater compared to PIKfyve^fl/fl,Aq‐Cre−^ mice (by ~30%) and these differences were with high statistical significance (Fig. 4C and F). The reproducibility of dysregulated systemic glucose tolerance and insulin resistance in the two mouse models is consistent with the notion that the PIKfyve reduction in the brain as observed in the PIKfyve^fl/fl,aP2‐Cre+^ mice may not contribute to the metabolic abnormalities manifested by the PIKfyve^fl/fl,aP2‐Cre+^ mice.

### Normal adiponectin but increased serum FFA in PIKfyve^fl/fl,Aq‐Cre+^ mice

**Figure 5:**
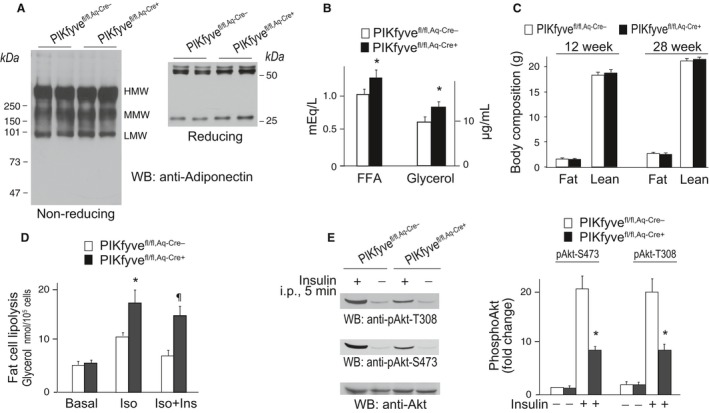
Increased serum FFA and glycerol but not adiponectin in PIKfyve^fl/fl,Aq‐Cre+^ mice along with elevated fat cell lipolysis and insulin resistance. (A) SDS‐PAGE of serum from 9‐month‐old PIKfyve^fl/fl,Aq‐Cre+^ and PIKfyve^fl/fl,Aq‐Cre−^ female mice was performed at reducing and nonreducing conditions, followed by WB with anti‐adiponectin antibodies. Shown are chemiluminescence detections from representative experiments with two females/genotype out of three independent determinations. (B) Glycerol and FFA were measured in serum of 9‐month‐old female mice following 4 h fasting; *n* = 6/group. *P <* *0.05 versus control PIKfyve^fl/fl,Aq‐Cre−^ mice. (C) in vivo body composition by EcoMRI of total fat and total lean mass in male mice at the indicated age (*n* = 6–7/group); (D) subsequent to 4 h fasting, fat cells freshly isolated from perigonadal fat of female mice (30‐week old) of both genotypes were subjected to lipolysis assay. Lipolysis was induced by isoproterenol (Iso, 25 nmol/L) and inhibited with physiological concentration of insulin (Ins, 17 nmol/L) as indicated. Glycerol concentrations were measured at 540 nm. *P <* *0.05, increased Iso‐triggered lipolysis versus respective basal; ^¶^ *P *<* *0.05, blunted antilipolyses by Ins versus control PIKfyve^fl/fl,Aq‐Cre−^ mice; *n* = 4/group. (E) Insulin (5 U/kg) or saline was administered i.p. for 5 min in 14 h fasted 40‐week‐old control and mutant male mice with comparable body weights but a trend for epididymal fat increases (by ~12%). RIPA ^2+^ lysates of epididymal fat were subjected to WB (75 *μ*g protein) with phosphoS473 Akt, phosphoT308 or total Akt antibodies. Shown are chemiluminescence detections from a representative experiment with two mice/group out of two independent determinations (*n* = 4). Quantified Akt phosphorylation in PIKfyve^fl/fl,Aq‐Cre+^ fat (right panel) shows significant reduction versus PIKfyve^fl/fl,Aq‐Cre−^; *P <* *0.05.

PIKfyve controls proper performance of a number of membrane trafficking pathways, including exocytosis or secretion of a plethora of ion channels, metabolite transporters, and other active molecules (Shisheva 2012). Therefore, we hypothesized that one mechanism by which PIKfyve deficiency in fat may trigger systemic glucose intolerance and insulin resistance is associated with reduced plasma levels of adiponectin, a hormone predominantly secreted by the fat tissue (Halberg et al. 2008). Reduced plasma concentration of adiponectin, and particularly that of high molecular weight (HMW) forms, has been previously shown to accelerate insulin resistance (Pajvani et al. 2004). However, as illustrated in Figure 5A, our examination of the different forms of serum adiponectin by SDS‐PAGE under reducing or nonreducing conditions and immunoblotting with adiponectin antibodies failed to reveal changes in PIKfyve^fl/fl,Aq‐Cre+^ versus PIKfyve^fl/fl,Aq‐Cre−^ mice. Next, as increased circulating levels of FFA could powerfully dysregulate systemic glucose homeostasis by decreasing insulin sensitivity of other metabolic organs (Guilherme et al. 2008; Kusminski et al. 2009), we assessed the fasting levels of circulating FFA and glycerol in 9‐month‐old PIKfyve^fl/fl,Aq‐Cre+^ and PIKfyve^fl/fl,Aq‐Cre−^ mice. Intriguingly, we found that serum FFA were markedly increased in the PIKfyve^fl/fl,Aq‐Cre+^ versus PIKfyve^fl/fl,Aq‐Cre−^ mice (Fig. 5B). These data indicate that the metabolic defects in PIKfyve^fl/fl,Aq‐Cre+^ could result in part from increased levels of circulating FFA.

### Increased lipolysis and blunted antilipolysis by insulin in fat cells from PIKfyve^fl/fl,Aq‐Cre+^ mice

Elevated circulating FFA levels are often associated with increased fat mass. It is thus possible that whereas PIKfyve^fl/fl,Aq‐Cre+^ mice had body weight similar to littermate controls (Fig. 2A and E) they could have exhibited a relative expansion of adipose mass. Our analysis of total fat and lean mass by noninvasive EchoMRI in young‐adult and mature‐adult mice did not detect changes in adiposity of PIKfyve^fl/fl,Aq‐Cre+^ mice (Fig. 5C). Intriguingly, the perigonadal fat dissected from males or females at 9 months of age showed a trend for an increase by 5–12% in the mutant mice of both genders, but this difference did not reach statistical significance. Thus, whereas the trend for fat mass increases might, in part, underlie the elevated circulating FFA, it probably does not fully explain the defect.

Another cellular mechanism that could account for the observed greater levels of circulating FFA in PIKfyve^fl/fl,Aq‐Cre+^ mice is increased lipolytic activity of fat cells (McGarry 2002). Consistently, we found that fat cells freshly isolated from perigonadal fat of PIKfyve^fl/fl,Aq‐Cre+^ mice displayed markedly elevated TG hydrolysis versus PIKfyve^fl/fl^ control mice upon *β*‐adrenergic receptor stimulation (isoproterenol) but the basal lipolysis was unaltered (Fig. 5D). Because insulin prominently inhibits lipolysis, we assessed its ability to counteract the lipolysis. As illustrated in Figure 5D, fat cells from of PIKfyve^fl/fl,Aq‐Cre+^ mice displayed blunted ability of insulin to restrain isoproterenol‐induced lipolysis compared to control PIKfyve^fl/fl,Aq‐Cre−^ mice.

The dampened antilipolytic activity of insulin in the PIKfyve^fl/fl,Aq‐Cre+^ fat cells suggests that the latter might be insulin resistant. A major molecular event of the insulin signaling cascade is Akt activation, which mediates many insulin cellular responses, including antilipolysis and glucose entry (Huang and Czech 2007; Choi et al. 2010). Studies in cultured 3T3L1 adipocytes have revealed that reduced PIKfyve protein or enzymatic activity dampens insulin‐induced Akt phosphorylation (Ikonomov et al. 2002, 2007; Shisheva 2008a), with the PIKfyve product PtdIns5P found to be a potent activator (Pendaries et al. 2006; Ramel et al. 2009; Jones et al. 2013). Therefore, to reveal whether adipose tissue PIKfyve/PtdIns5P ablation affects the in vivo Akt phosphorylation in fat, we performed immunoblotting with phosphoAkt antibodies of adipose tissue lysates derived from insulin‐ or saline‐injected PIKfyve^fl/fl,Aq‐Cre+^ and PIKfyve^fl/fl^ mice. As illustrated in Figure 5E, whereas 5 min post injection the AktS473 and AktT308 sites were profoundly phosphorylated in fat of control mice, their phosphorylation was greatly suppressed in fat from PIKfyve^fl/fl,Aq‐Cre+^ mice. These data indicate blunted Akt phosphorylation in fat tissue upon disruption of adipose *Pikfyve*, consistent with the requirement of adipose PIKfyve and its lipid products for efficient Akt phosphorylation by insulin.

### Severe mammary gland phenotype by aP2‐Cre‐ but not by Aq‐Cre‐driven PIKfyve deficiency

While breeding the aP2‐Cre mouse line, we made the surprising observation that whereas completely fertile, giving birth to a similar number of pups as the heterozygous PIKfyve^fl/wt,aP2‐Cre+^ or control PIKfyve^fl/fl^ littermates, the PIKfyve^fl/fl,aP2‐Cre+^ mothers failed to nurse their pups either from first (Table 1) or second and third pregnancies (in each, *n* = 3 PIKfyve^fl/fl,aP2‐Cre+^). However, the offspring from PIKfyve^fl/fl^ females and PIKfyve^fl/fl,aP2‐Cre+^ males developed without problems. The pups born to female PIKfyve^fl/fl,aP2‐Cre+^ dams died 6 – 96 h postpartum. There was no milk in pups’ stomachs, visible through their translucent bodies, suggesting a failure of PIKfyve^fl/fl,aP2‐Cre+^ dams to lactate. Maternal instincts seemed preserved as judged by the fact that PIKfyve^fl/fl,aP2‐Cre+^ dams built a nest days before parturition and initially lay on top of the litter, but later, when anxiety took over, they abandoned the nest even when the pups were still alive (Movie S1). Because pups born to PIKfyve^fl/wt,aP2‐Cre+^ dams were nurtured normally (Table 1), an adverse effect of the Cre transgene was ruled out (Palmer et al. 2006; Robinson and Hennighausen 2011). Likewise, PIKfyve^fl/fl,Aq‐Cre+^ dams, in which adipose PIKfyve was even more efficiently eliminated than in PIKfyve^fl/fl,aP2‐Cre+^ (Figs. 1, 3) successfully nursed their litters (Table 1), thus, dismissing the possibility for PIKfyve deficiency in adipose tissue underlying the mammary gland defect during pregnancy.

**Figure 6:**
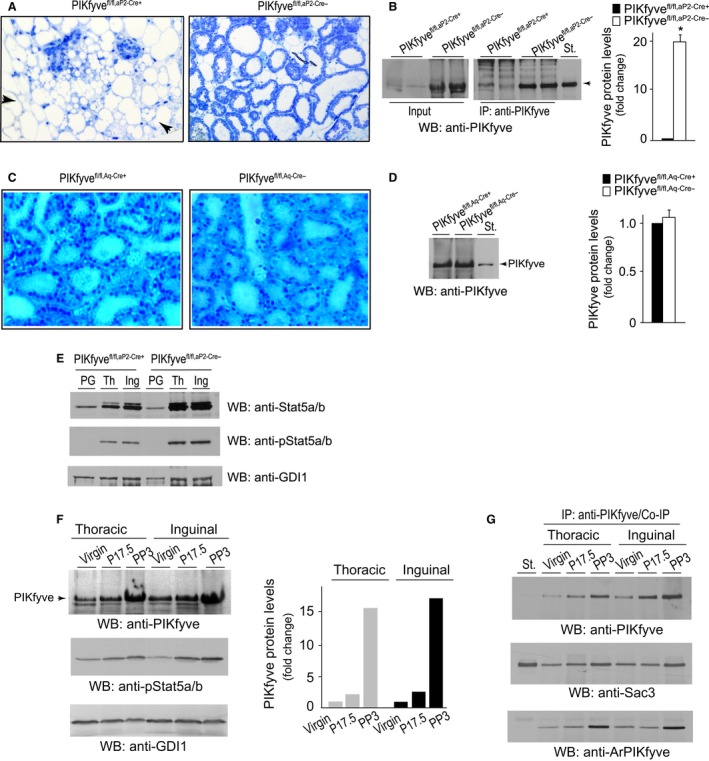
*Pikfyve* disruption by aP2‐Cre, but not by Aq‐Cre, causes severe mammary phenotype. (A) Histology of formalin‐fixed plastic‐embedded stained sections of mammary glands (#4, inguinal) derived from 9‐week‐old PP3 PIKfyve^fl/fl,^ ^aP^ ^2‐Cre+^ and PIKfyve^fl/fl,^ ^aP^ ^2‐Cre−^ primiparous females. Apparent is severely underdeveloped gland with few lobuloalveolar structures within the bulk of mammary fat cells in PIKfyve^fl/fl,^ ^aP^ ^2‐Cre+^ dams (arrows). Alveolar lobuli of control PIKfyve^fl/fl,^ ^aP^ ^2‐Cre−^ glands are well‐expanded and milk‐filled. (B) Clarified RIPA ^+^ lysates (150 *μ*g protein) from mammary glands dissected from 9‐week old PP3 PIKfyve^fl/fl,^ ^aP^ ^2‐Cre+^ and PIKfyve^fl/fl,^ ^aP^ ^2‐Cre−^ primiparas were examined by WB with anti‐PIKfyve antibodies directly (left panel) or subsequent to IP with anti‐PIKfyve antibodies (right panel). Shown are chemiluminescence detections from a representative experiment with two mice/genotype of three determinations. Graph, quantified changes in PIKfyve levels between the two genotypes. *P <* *0.001, *n* = 3. (C) Histology of formalin‐fixed plastic‐embedded stained sections of mammary glands (#4, inguinal) derived from 9‐week‐old PP3 PIKfyve^fl/fl,Aq‐Cre+^ and PIKfyve^fl/fl,Aq‐Cre−^ primiparas, showing similar lobuloalveolar structures in the two genotypes. (D) Clarified RIPA ^+^ lysates (150 *μ*g protein), derived from mammary glands dissected from 9‐week‐old PP3 PIKfyve^fl/fl,Aq‐Cre+^ and PIKfyve^fl/fl,Aq‐Cre−^ primiparous sisters were examined by WB with anti‐PIKfyve antibodies. Shown is a chemiluminescence detection from a representative experiment out of three with similar results and quantification. (E) Clarified RIPA ^2+^ lysates (40 *μ*g protein) obtained from perigonadal fat (PG) or thoracic (Th) and inguinal (Ing) mammary glands dissected from 20‐week‐old PP3 PIKfyve^fl/fl,^ ^aP^ ^2‐Cre+^ and PIKfyve^fl/fl,^ ^aP^ ^2‐Cre−^ primiparous sisters were examined by WB with anti‐Stat5 and anti‐phospho‐Stat5 antibodies with a stripping step in between. The blot was reprobed with anti‐GDI1 to normalize for loading. Shown are chemiluminescence detections from a representative experiment out of two with similar results. (F) RIPA ^2+^ lysates were obtained from three PIKfyve^fl/fl^ littermate females at the stage of virgin, pregnancy day 17.5 (P17.5) and postpartum day 3 (PP3). Clarified lysates were analyzed by WB (150 *μ*g protein) with the indicated antibodies (left panel). Shown are chemiluminescence detections from a representative experiment probed with anti‐PIKfyve (the upper half of the membrane) and anti‐phospho‐Stat5 antibodies (the lower half of the membrane). The lower half of the membrane was reprobed with anti‐GDI1 to normalize for loading. The increase in PIKfyve levels is quantified from two independent experiments with similar results. (G) Lysates (600 *μ*g) obtained as in *F* were analyzed by immunoprecipitation with anti‐PIKfyve antibodies to quantify the interaction partners Sac3 and ArPIKfyve and their association with PIKfyve. Shown are chemiluminescence detections from a representative IP experiment probed with the indicated antibodies subsequent to stripping. St., in B, D, & G, PIKfyve, Sac3, or ArPIKfyve standards from lysates of COS7 cells overexpressing the proteins.

The ability to nurse correlates with the extent of alveolar development and differentiation. Remarkably, at postpartum day 3 (PP3) both thoracic and inguinal glands in PIKfyve^fl/fl,aP2‐Cre+^ dams weighed markedly less (by 2.4 ± 0.5 fold and 6.9 ± 0.9 fold, respectively, and Figure S1) compared to those in PIKfyve^fl/fl,aP2‐Cre−^ littermates, suggesting defective alveolar growth and differentiation during pregnancy. Strikingly, histological examination of mammary glands in PP3 PIKfyve^fl/fl,aP2‐Cre+^ mothers revealed a severely undeveloped lobulo‐alveolar system occurring at only ~15% density of the mammary fat pad and predominance of mammary adipocytes (Fig. 6A). The sparse alveoli‐like structures in the PIKfyve^fl/fl,aP2‐Cre+^ glands were also morphologically different in that they did not exhibit open lumen and were practically milk‐empty. In contrast, postpartum glands of control PIKfyve^fl/fl,aP2‐Cre−^ females exhibited fully developed and distended milk‐filled alveoli, occupying the entire mammary fat pad (Fig. 6A). Remarkably, western blot analysis of mammary gland lysates derived from postpartum PIKfyve^fl/fl,aP2‐Cre+^ mice revealed practically undetectable levels of PIKfyve, a result further confirmed through concentrating the PIKfyve protein on anti‐PIKfyve antibodies and analyzing by immunoprecipitation (Fig. 6B). However, there were exceptionally high levels of PIKfyve levels in PP3 PIKfyve^fl/fl,aP2‐Cre−^ mammary glands (Fig. 6B) not seen thus far in any other tissue. Quantification of a dilution series of mammary gland lysates by Western blotting revealed >20‐fold increase of PIKfyve levels in PP3 PIKfyve^fl/fl,aP2‐Cre−^ versus PP3 PIKfyve^fl/fl,aP2‐Cre+^ dams. Notably, similar histology and Western blot analyses in mammary glands showed no substantial differences between PP3 PIKfyve^fl/fl,Aq‐Cre+^ and PIKfyve^fl/fl,Aq‐Cre−^ mothers with respect to morphological appearance and density of the lobulo‐alveolar system within the mammary stroma (Fig. 6C) as well as PIKfyve protein levels (Fig. 6D).

Stat5a, a transcription factor that is phosphorylated downstream of the prolactin receptor (PrlR) activation, is the principal mediator of mammary gland development (Liu et al. 1997; Cui et al. 2004). Whereas it is expressed in all tissue, both total and phosphorylated Stat5 levels profoundly increase in mammary glands during late pregnancy and lactation. To reveal if abnormal Stat5 levels and/or activation occur in PIKfyve^fl/fl,aP2‐Cre+^ dams upon parturition, we performed Western blotting of mammary gland lysates with antibodies for total and phospho‐Stat5a/b. As illustrated in Figure 6E, there was a profound suppression in the surge of phospho‐ and total Stat5 levels in both thoracic and inguinal glands from PP3 PIKfyve^fl/fl,aP2‐Cre+^ versus littermate PIKfyve^fl/fl,aP2‐Cre−^ mothers. Conversely, perigonadal Stat5 remained similar in mutant and control dams (Fig. 6E).

The striking increase in PIKfyve protein levels seen in lactating mammary glands of PIKfyve^fl/fl,aP2‐Cre−^ or PIKfyve^fl/fl,Aq‐Cre+^ dams suggests the possibility that PIKfyve expression is markedly upregulated under the stimulus of pregnancy. We addressed this prediction by Western blotting with and without PIKfyve immunoprecipitation of lysates derived from both thoracic and inguinal mammary glands in nulliparous virgin, pregnant or postpartum day 3 lactating control PIKfyve^fl/fl^ littermate females. This analysis revealed a slight PIKfyve increase in late pregnancy (by 2.5‐fold), but massive elevation after parturition (>15‐fold), concomitant with increased Stat5a/b activation (Fig. 6F). The surge in PIKfyve levels was paralleled by elevation in the PIKfyve complex, evidenced herein by the increased Sac3 phosphatase and ArPIKfyve scaffolding protein recovered in PIKfyve immunoprecipitates (Fig. 6G). Taken together, these data indicate that the inability of PIKfyve^fl/fl,aP2‐Cre+^ to respond to the stimulus of pregnancy is associated with arrested epithelial cell proliferation and lack of concomitant surge in PIKfyve levels. Our data are consistent with the notion that PIKfyve function is required in mammary epithelial differentiation and lactogenesis either as part of the PrlR‐Jak2‐Stat5 signaling pathway or as a parallel pathway.

### Prolactin upregulates PIKfyve in an ex vivo mammary gland model of lactation

**Figure 7:**
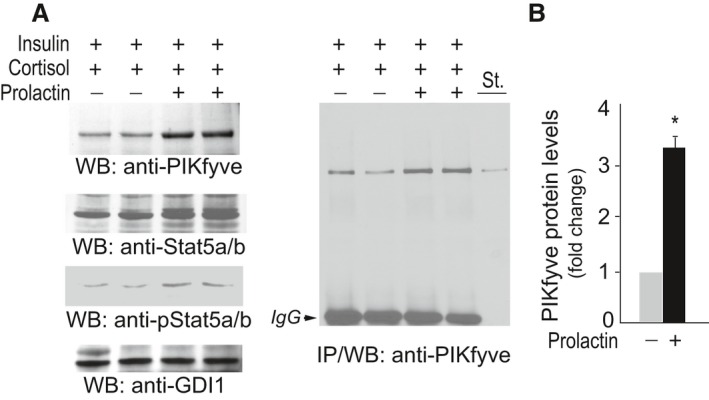
PIKfyve is upregulated by PrlR activation in mammary gland explants of midpregnant mice. (A) Mammary gland explants derived from 6 to 8 midpregnant mice were cultured for 24 h with 1 *μ*g/mL insulin and 10^−7^ mol/L cortisol. Incubation was then continued for additional 24 h after addition of Prl (1 *μ*g/mL) where indicated. Clarified RIPA ^2+^ lysates from duplicate cultures were analyzed by WB (40 *μ*g protein) with the indicated antibodies (left panel) and immunoprecipitation (IP) with the PIKfyve antibodies (right panel). St., lysates from COS7 cells overexpressing PIKfyve. Shown are chemiluminescence detections from representative WB and IP out of four independent experiments in duplicates. The membrane was reprobed with anti‐GDI1 to normalize for loading. (B) Quantification of the PIKfyve increases upon PrlR stimulation. *P <* *0.001 versus prolactin nonstimulated mammary explants.

To delineate if PIKfyve's role in mammary gland development during pregnancy and lactation is mechanistically linked with the PrlR signaling pathway, we used mammary gland explants derived from midpregnant female mice, an ex vivo system that we have previously developed and characterized with respect to prolactin‐induced milk protein production (Rillema and Schneider‐Kuznia 1980; Rillema 1994). Subsequent to 24 h culturing in media containing insulin and cortisol, mammary gland explants were treated with or without prolactin (Prl) for additional 24 h. As illustrated in Figure 7A and B, Prl profoundly increased PIKfyve protein levels as revealed by western blotting with anti‐PIKfyve antibodies, with or without prior immunoprecipitation. The upregulation of PIKfyve coincided with a rise in total and phospho‐Stat5a/b (Fig. 7A). These data indicate that the PIKfyve pathway is activated downstream of PrlR stimulation and suggest that PIKfyve is required to confer the response to Prl during lactation.

## Discussion

### Adipose PIKfyve is essential for glucose homeostasis

Due to the ability to store lipids and function as an endocrine organ, the adipose tissue controls insulin sensitivity of other metabolic organs, such as liver, muscle, and pancreas, which in turn, maintain glucose, and lipid homeostasis (Herman and Kahn 2006; Halberg et al. 2008). Therefore, pathophysiological conditions affecting fat tissue, including insulin resistance, ultimately impair systemic glucose tolerance and insulin sensitivity, leading to obesity‐related metabolic diseases. Whereas cell studies with PIKfyve protein knockdown or inhibition of the lipid kinase activity have established reduced insulin responsiveness of glucose uptake in 3T3L1 adipocytes (Ikonomov et al. 2007, 2009b; Shisheva 2008a; Koumanov et al. 2012), whether adipose tissue PIKfyve deficiency will impact systemic glucose homeostasis remains unknown. In this study, we addressed this question by taking advantage of a recently generated mouse model, in which the *Pikfyve* gene can be deleted in a tissue‐specific manner by the Cre‐loxP approach (Ikonomov et al. 2011, 2013). We developed two new mouse lines with Cre‐mediated inactivation driven by the promoters of the aP2/FABP4 or adiponectin genes, both of which confer recombination in adipose tissue, though with a different efficacy and specificity (Lee et al. 2013; Jeffery et al. 2014). We report here for the first time that mice with adipose PIKfyve deficiency exhibit systemic glucose intolerance and insulin resistance as judged by the accelerated rise and blunted fall of blood glucose in response to a systemic load of glucose and insulin, respectively (Figs. 2, 4).

The usage of two independent promoters for adipose‐specific gene disruption supports our conclusion that the primary defect underlying the whole‐body glucose intolerance and insulin resistance in PIKfyve^fl/fl,aP2‐Cre+^ and PIKfyve^fl/fl,Aq‐Cre+^ mice is the PIKfyve deficiency specifically targeted in adipose tissue. Data interpretation based solely on the aP2‐Cre‐driven recombination of adipose *Pikfyve* could have posed a significant level of uncertainty, as we, like others (Urs et al. 2006; Martens et al. 2010; Heffner et al. 2012; Zhang et al. 2012), have noted substantial PIKfyve ablation in the brain (Fig. 1). Disrupted neuronal function on its own could impair whole‐body response to a systemic glucose load and affect overall physiological control of blood glucose (Parton et al. 2007; Osundiji and Evans 2011). Therefore, to avoid confounding interpretations due to neuronal PIKfyve loss, we focused on the mechanism(s) by which adipose PIKfyve triggers metabolic dysfunction in the PIKfyve^fl/fl,Aq‐Cre+^ model that, in addition to higher specificity, exhibited also greater efficacy of PIKfyve ablation in fat tissue than the PIKfyve^fl/fl,aP2‐Cre+^ mice (Figs. 1, 3). Because the plasma adiponectin concentration is inversely associated with insulin resistance or T2D and because PIKfyve is a powerful regulator of exocytosis (Shetty et al. 2009; Shisheva 2012), we envisioned reduced adiponectin secretion by adipose tissue to underlie the observed metabolic defects. However, whereas such changes were not apparent (Fig. 5A), we found an unanticipated rise in circulating levels of FFA, with corresponding increases in serum glycerol (Fig. 5B). High circulating FFA have long been known to impair insulin sensitivity of muscle or liver and blunt insulin secretion by pancreatic beta cells, resulting in hyperglycemia (Haber et al. 2003; Herman and Kahn 2006; Jensen 2006; Ye 2007; Rhodes et al. 2013). It will be important in future studies to reveal the relative contribution for each of these tissues to the systemic glucose intolerance observed in PIKfyve^fl/fl,Aq‐Cre+^ mice.

An important insight in our study is the delineation of the cellular mechanism by which the loss of adipose PIKfyve causes elevation of circulating FFA. Thus, our findings for markedly augmented lipolytic activity in PIKfyve^fl/fl,Aq‐Cre+^ fat cells upon *β*‐adrenergic receptor stimulation (Fig. 5D) corroborate the notion that adipose PIKfyve deficiency impairs fat tissue TG storage, resulting in elevation of serum FFA levels. A potential signaling mechanism for accelerated fasted fat cell lipolysis may involve mTORC1 (mammalian target of rapamycin complex 1), whose reduced activation promotes TG hydrolysis (Chakrabarti et al. 2010; Soliman et al. 2010). Reportedly the PIKfyve lipid product PtdIns(3,5)P_2_ activates mTORC1 (Bridges et al. 2012), an observation consistent with the notion that the higher TG hydrolysis observed in the PIKfyve^fl/fl,Aq‐Cre+^ fat cells could be due at least in part to reduced mTOR1 activation. Furthermore, in the face of severe fat‐cell insulin resistance in PIKfyve^fl/fl,Aq‐Cre+^ mice, as evidenced in our study by the profound reduction in Akt activation in response to intraperitoneal injection of insulin, we observed reduced ability of insulin to restrain *β*‐adrenergic receptor‐activated lipolysis (Fig. 5D and E). Sustained insulin activation of Akt is negatively regulated by the PP2A phosphatase (Ugi et al. 2004; Liao and Hung 2010; Toker and Marmiroli 2014). Intriguingly, the PIKfyve lipid product PtdIns5P is a powerful suppressor of the PP2A activity (Ramel et al. 2009), which may explain the lower levels of phospho‐Akt under adipose PIKfyve deficiency. Reduced phosphoAkt may further blunt the mTORC1 activation (Lamming and Sabatini 2013; Chakrabarti and Kandror 2015) to deepen accelerated lipolysis in PIKfyve^fl/fl,Aq‐Cre+^ fat cells. Furthermore, in the presence of adipocyte insulin resistance, effective re‐esterification of FFA is likely to be hampered, as the source for the required supply of glycerol 3‐phosphate is the insulin‐sensitive glycolysis and/or glyconeogenesis, thus, further elevating FFA (Herman and Kahn 2006; Kusminski et al. 2009). It should be noted, however, that the observed elevation of serum FFA and increased fat cell lipolytic rate in PIKfyve^fl/fl,Aq‐Cre+^ mice may not be the only mechanisms by which adipose PIKfyve deficiency impairs whole‐body glucose homeostasis. This prediction is corroborated by our secretome analysis (Shisheva and Chen, in preparation), revealing increased secretion by the PIKfyve^fl/fl,Aq‐Cre+^ fat cells of a number of proinflammatory factors such as plasminogen activator inhibitor 1, haptoglobin, and thrombospondin1, which contribute to insulin resistance (Deng and Scherer 2010; Jung and Choi 2014).

### Unexpected significance of PIKfyve to mammogenesis and lactogenesis

Whereas the aP2 promoter may be less adipose‐specific than originally thought, its off‐target expression in various tissues and organs has helped in disclosing important and unexpected functions or mechanisms (Zhang et al. 2012; Xiang et al. 2013). Our study provides another paradigm for such an unanticipated mechanism. Thus, we revealed a critical role for PIKfyve in mammary gland development, which emerged as an unintended consequence of aP2‐Cre driven *pikfyve* recombination in off‐target tissues. Our observation for severely reduced lobuloalveolar growth in PIKfyve^fl/fl,aP2‐Cre+^ females and the unconditional failure of dams to produce milk and nurse (Fig. 6A and Table 1) establishes an unforeseen function of PIKfyve in mammary epithelial differentiation, proliferation, and lactogenesis. Because the PIKfyve^fl/fl,Aq‐Cre+^ females did not have such a defect (Table 1), ablation of adipose tissue *Pikfyve* as an underlying cause is highly improbable. Likewise, systemic heterozygous PIKfyve^WT/KO^ mice, with one half of the wild type gene/protein in all tissues (Ikonomov et al. 2011), readily nurse their offspring. These data suggest that the defect in the PIKfyve^fl/fl,aP2‐Cre+^ females is due to a highly efficient off‐target *Pikfyve* recombination. Because recent data indicate high promiscuity of aP2‐Cre expression at both pre‐ and postnatal stages (Heffner et al. 2012), the off‐target sites of *Pikfyve* inactivation triggering impaired mammogenesis was not addressed in our study. However, we surmised the mammary epithelial precursor cells as an off‐target candidate. Their propagation and differentiation during pregnancy and lactation is likely to require PIKfyve, as suggested by our observations for excessive increases of the mammary epithelium PIKfyve after parturition (Fig. 6C, F). The embryonic lethality of the global *pikfyve* disruption occurring as early as the preimplantation stage of the differentiation/developmental program due, in part, to arrested DNA synthesis/cell division (Ikonomov et al. 2011) also supports this prediction. Future studies using mammary gland‐specific promoters for *Pikfyve* excision will enlighten this issue.

A failure of mammary gland development and lactation during pregnancy, as observed in our PIKfyve^fl/fl,aP2‐Cre+^ model, is well‐characterized in female mice lacking individual members of the PrlR signaling cascade, including PrlR, Jak2 (Janus kinase 2), Stat5, and Stat5 regulators (Wagner and Rui 2008). Because PIKfyve was diminished in the brain of PIKfyve^fl/fl,aP2‐Cre+^ females (Fig. 1B), one might link the mammary gland defect with presumable Prl deficiency. Whereas reasonable, such a possibility seems unlikely in light of data indicating major infertility problems in female mice upon *Prl* inactivation (Horseman et al. 1997), contrary to our PIKfyve^fl/fl,aP2‐Cre+^ females, which readily conceived and produced normal litter sizes (Table 1). These observations together with other data in our study suggest that the PIKfyve pathway and the PrlR‐Jak2‐Stat5 signaling are interconnected. For example, concomitant with Stat5 activation, PIKfyve is also greatly upregulated in mammary epithelium subsequent to PrlR activation by the stimulus of pregnancy or by prolactin treatment of mammary gland explants (Figs. 6F, 7). Concordantly, postpartum PIKfyve^fl/fl,aP2‐Cre+^ females exhibited a marked reduction in Stat5 expression/activation in mammary glands (Fig. 6E). It should be emphasized, however, that in addition to PIKfyve deficiency, a lactation failure could also contribute to the deregulated Stat5 in PIKfyve^fl/fl,aP2‐Cre+^ females, thus making the relative contribution of each defect an important objective for future studies. Notwithstanding, our data in the mammary gland explants (Fig. 7) clearly indicate that PIKfyve is among the genes whose expression is regulated by PrlR activation. It is likely that PIKfyve, similar to the PI3K‐Akt in PrlR‐Jak2‐Stat5 signaling, may function as an upstream regulator of Stat5, downstream effector of Stat5, or both (Chen et al. 2010, 2012; Schmidt et al. 2014), which remains to be clarified in future research. In any case, our data indicate for the first time that PIKfyve and its lipid products, in addition to the signal‐transduction cascade of insulin (Sbrissa et al. 2004b; Ikonomov et al. 2007; Shisheva 2008a; Sarkes and Rameh 2010; Bridges et al. 2012; Koumanov et al. 2012), are also implicated in that of Prl.

### Summary

The data presented herein reveal that adipose tissue PIKfyve is a key regulator of systemic glucose homeostasis. The usage of the aP2 promoter for *Pikfyve* ablation unraveled an unanticipated requirement for PIKfyve in mammary gland development and differentiation during pregnancy, downstream of prolactin‐receptor signaling.

## Conflict of Interest

None declared.

## Supporting information




**Figure S1.** Littermates of the indicated genotype at postpartum day 3. Larger inguinal mammary glands (arrowheads) in the PIKfyve^fl/fl,aP2‐Cre−^ versus PIKfyve^fl/fl,aP2‐Cre+^ dams.

**Movie S1.** PIKfyve^fl/fl,aP2‐Cre+^ dams built a nest days before parturition and initially lay on top of the litter, but later, when anxiety took over, they abandoned the nest even when the pups were still alive.
