# Supplementary material for: Unexpected severe consequences of Pikfyve deletion by aP2‐ or Aq‐promoter‐driven Cre expression for glucose homeostasis and mammary gland development
Source: Physiol Rep. 2016 Jun 7;4(11):e12812. doi: 10.14814/phy2.12812 (PMC4908490; doi:10.14814/phy2.12812)
Supplement: Supplementary file 1 — Figure S1. Littermates of the indicated genotype at postpartum day 3. Larger inguinal mammary glands (arrowheads) in the PIKfyvefl/fl,aP2‐Cre− versus PIKfyvefl/fl,aP2‐Cre+ dams. [file PHY2-4-e12812-s001.docx]

**Supplementary Material**

**Unexpected Severe Consequences of *Pikfyve* Deletion by aP2- or Aq-promoter-driven Cre Expression for Glucose Homeostasis and Mammary Gland Development**

Ognian C. Ikonomov#, Diego Sbrissa#, Khortnal Delvecchio, James Rillema, and Assia Shisheva*

Suppl. Fig. 1. Littermates of the indicated genotype at post-partum day 3. Larger inguinal mammary glands (arrowheads) in the PIKfyve^fl/fl,aP2-Cre-^ vs. PIKfyve^fl/fl,aP2-Cre+^  dams.

Suppl. Movie 1: PIKfyve^fl/fl,aP2-Cre+^ dams built a nest days before parturition and initially lay on top of the litter, but later, when anxiety took over, they abandoned the nest even when the pups were still alive.
